# Failure of CD4 T-Cells to Respond to Liver-Derived Antigen and to Provide Help to CD8 T-Cells

**DOI:** 10.1371/journal.pone.0021847

**Published:** 2011-07-14

**Authors:** Katja Derkow, Anja Müller, Ira Eickmeier, Daniel Seidel, Marcos Vicinius Rust Moreira, Nils Kruse, Katja Klugewitz, Justine Mintern, Bertram Wiedenmann, Eckart Schott

**Affiliations:** 1 Department of Hepatology and Gastroenterology, Charité Universitätsmedizin Berlin, Campus Virchow-Klinikum (CVK), Berlin, Germany; 2 Department of Neurology, Charité Universitätsmedizin Berlin, Campus Charité Mitte (CCM), Berlin, Germany; 3 Department of Gastroenterology and Infectious Diseases, Charité Universitätsmedizin Berlin, Campus Benjamin Franklin (CBF), Berlin, Germany; 4 Department of Microbiology and Immunology, University of Melbourne, Victoria, Australia; Centre de Recherche Public de la Santé (CRP-Santé), Luxembourg

## Abstract

CD4 T-cell help is required for the induction of efficient CD8 T-cells responses and the generation of memory cells. Lack of CD4 T-cell help may contribute to an exhausted CD8 phenotype and viral persistence. Little is known about priming of CD4 T-cells by liver-derived antigen. We used TF-OVA mice expressing ovalbumin in hepatocytes to investigate CD4 T-cell priming by liver-derived antigen and the impact of CD4 T-cell help on CD8 T-cell function. Naïve and effector CD4 T-cells specific for ovalbumin were transferred into TF-OVA mice alone or together with naïve ovalbumin-specific CD8 T-cells. T-cell activation and function were analyzed. CD4 T-cells ignored antigen presented by liver antigen-presenting cells (APCs) *in vitro* and *in vivo* but were primed in the liver-draining lymph node and the spleen. No priming occurred in the absence of bone-marrow derived APCs capable of presenting ovalbumin *in vivo*. CD4 T-cells primed in TF-OVA mice displayed defective Th1-effector function and caused no liver damage. CD4 T-cells were not required for the induction of hepatitis by CD8 T-cells. Th1-effector but not naïve CD4 T-cells augmented the severity of liver injury caused by CD8 T-cells. Our data demonstrate that CD4 T-cells fail to respond to liver-derived antigen presented by liver APCs and develop defective effector function after priming in lymph nodes and spleen. The lack of CD4 T-cell help may be responsible for insufficient CD8 T-cell function against hepatic antigens.

## Introduction

While activation of CD8 T-cells proceeds in the absence of CD4 T-cells, the latter's help is crucial for the development of long lasting memory. Although the primary response of CD8 T-cells activated in the absence or presence of CD4 T-cells is comparable, CD8 T-cells generated in the absence of CD4 T-cell help display poor recall responses and produce little cytokines upon their second encounter with antigen [1], [2]. CD8 T-cells primed in the absence of CD4 T-cell help display an exhausted phenotype characterized by high levels of PD-1 [3]. This exhausted phenotype and the lack of CD4 T-cell help may contribute to the persistence of virus in chronic hepatitis. Indeed, lack of CD4 T-cells help may explain why CD8 T-cells fail to eradicate hepatitis C virus [4].

The liver represents a unique environment for antigen presentation because several populations of professional and non-professional antigen-presenting cells (APCs) reside there, and its environment in general skews T-cell responses towards tolerance (reviewed in [5]). In addition to the possibility of antigen presentation within the liver by resident dendritic cells (DCs) [6], Kupffer cells (KCs) [7], liver sinusoidal endothelial cells (LSECs) [8], and hepatocytes [9], the liver is also sampled by dendritic cells, which subsequently migrate to secondary lymphatic organs and present antigen there [10]. While CD4 T-cells recognize antigen obtained by phagocytosis and presented by APCs, CD8 T-cell responses are restricted to antigens derived from brake-down products within the target cell. However, through a mechanism termed cross-presentation, CD8 T-cells may also respond to exogenous or endogenous antigens presented by professional APCs after uptake and processing [11].

CD4 T-cells need to have access to the same antigen that is recognized by CD8 T-cells to be able to provide help. We and others have developed mouse models, in which a neoantigen is expressed in the liver and which allow the investigation of both CD8 and CD4 T-cell activation by this antigen [12], [13], [14], [15]. Using these models, it has become evident that CD8 T-cells primed in the liver by APCs acquire effector function rather than become tolerant. The ability of liver APCs to prime CD4 T-cells in an antigen specific manner is less well characterized. Expression of MHC-II molecules on hepatocytes triggers CD4 T-cell activation [16], but T-cells primed under these conditions are defective [17]. LSECs constitutively express MHC-II but it is still a matter of debate whether they are capable of stimulating CD4 T-cells [18], [19], [20], [21]. *In vitro* experiments demonstrate that CD4 T-cells primed by LSECs lack effector function [18] or display a regulatory phenotype [22]. CD4 T-cells primed by liver DCs *in vitro* proliferate, yet little Th1 effector cytokines are produced [23].

We have analyzed CD4 T-cell activation by liver-derived antigen *in vitro* and *in vivo* and investigated the role of CD4 T-cell help in a CD8 T-cell dependent model of immune-mediated liver injury.

## Results

### Failure of naïve CD4 T-cells to respond to liver-derived antigen *in vitro*

To determine the capability of specific populations of liver APC to activate CD4 T-cells, we purified DCs, KCs, or LSECs from the livers of TF-OVA mice, which express ovalbumin in hepatocytes. The endogenous antigen should be present in the different types of APCs, which acquire the antigen through phagocytosis of hepatocyte remnants. Therefore, incubation of OT-II CD4 or OT-I CD8 T-cells with specific APCs will test the APCs' ability to stimulate naïve T-cells with an antigen that is derived from an endogenous source and presented or cross-presented after processing.

**Figure 1:**
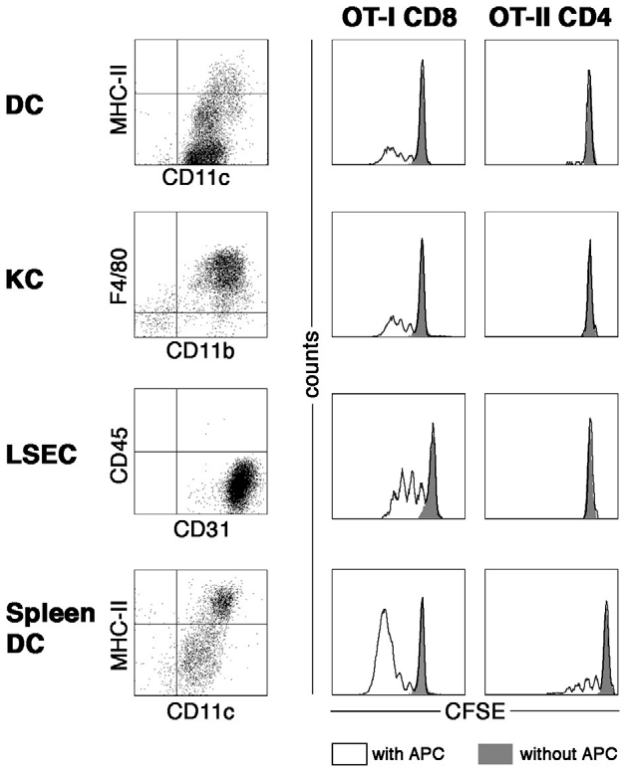
Priming of naive CD4 T-cells by liver-derived antigen *in vitro.* Dendritic cells (DC), Kupffer cells (KC) and liver sinusoidal cells (LSEC) were isolated from livers of TF-OVA mice, DCs were also isolated from spleens of TF-OVA mice. APCs (2×10^5^ cells) were allowed to settle for 24 h before addition of 1×10^5^ CFSE labeled T-cells. After three days of co-culture, cells were harvested, stained for CD8 or CD4, and proliferation of OT-I or OT-II T-cells was analyzed (black line) by CFSE dilution. OT-II and OT-I T-cells incubated alone were used as negative control (filled gray histogram). Representative results from n = 3–6 experiments are shown. Plots depict data gated on CD8^+^CFSE^+^ or CD4^+^CFSE^+^ cells.

OT-I T-cell proliferation was observed after incubation with DCs, KCs, or LSECs, indicating that liver-derived antigen had indeed been taken up and processed by the APCs (Fig. 1). Nevertheless, OT-II T-cells did not proliferate when incubated with either one of the APCs presenting liver-derived antigen. When liver APCs were incubated with exogenous ovalbumin beforehand, proliferation of CD4 T-cells was restored, indicating that APCs are in principle capable of processing antigen and activating CD4 T-cells (data not shown). DCs isolated from the spleens of TF-OVA mice induced proliferation in both OT-I and OT-II T-cells. These data indicate that liver APCs are incapable of stimulating naïve CD4 T-cells with liver-derived antigen *in vitro* although they do stimulate CD8 T-cells.

### Failure of naïve CD4 T-cells to respond to liver-derived antigen *in vivo*

**Figure 2:**
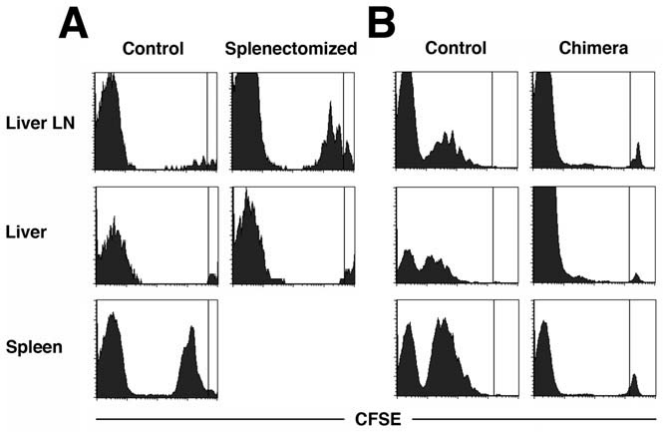
Priming of naive CD4 T-cells by liver-derived antigen *in vivo.* OT-II T-cells were purified from the lymph nodes and spleen of OT-II mice and labeled with CFSE. Four million cells were transferred intravenously into splenectomized TF-OVA mice (A), MHC-II^−/−^ → TF-OVA chimeras (B), or TF-OVA mice (control). Cells from the indicated organs were isolated 44 (A) or 68 hours (B) after cell transfer and analyzed for the presence of proliferating OT-II T-cells by detection of CFSE-dilution. All plots depict data gated on CD4^+^ cells. Events to the right of the vertical line represent the undivided population. Events at the far left of the plot represent unlabeled endogenous cells. Representative results from n = 4 bone-marrow chimeras and n = 4 control mice (A), and n = 6 splenectomized and n = 4 control mice (B) are shown.

Similar to the observed effects *in vitro*, no priming of naïve OT-II T-cells in the liver was observed *in vivo* [13]. Rather, activation occurred in spleen and liver-draining lymph node (Fig. 2A). Since the majority of CD4 T-cells were activated in the spleen, we transferred naïve OT-II T-cells into splenectomized TF-OVA mice. Priming of OT-II T-cells was observed in the liver draining lymph node but not in the liver in splenectomized TF-OVA mice. Our results imply that the spleen is dispensable for the priming of naïve CD4 T-cells and that APCs within the liver are insufficient to induce their priming *in vivo*, leaving the liver draining lymph node as the key location for priming of CD4 T-cells by liver-derived antigen.

To investigate more thoroughly the requirements for the priming of naïve CD4 T-cells by liver-derived antigen *in vivo*, we generated bone-marrow chimeras, in which professional APCs are incapable of presenting the antigen to OT-II T-cells. In TF-OVA mice reconstituted with bone marrow from MHC-II^−/−^ mice, no priming of OT-II T-cells was observed, even after a prolonged observation period of 68 h (Fig. 2B), indicating that professional bone-marrow derived APCs are required for the priming of naïve CD4 T-cells by liver-derived antigen. At the 68 h time point, proliferating CD4 T-cells were retrieved from the liver. The lack of proliferation at the earlier time point and the fact that only cells were retrieved that had divided multiple times suggest that these cells had migrated to the liver after activation in spleen and lymph nodes. CD4 T-cells retrieved from the liver exclusively belonged to a population that had undergone several rounds of proliferation in consistence with the liver's preference to recruit activated rather than naïve T-cells [24].

**Figure 3:**
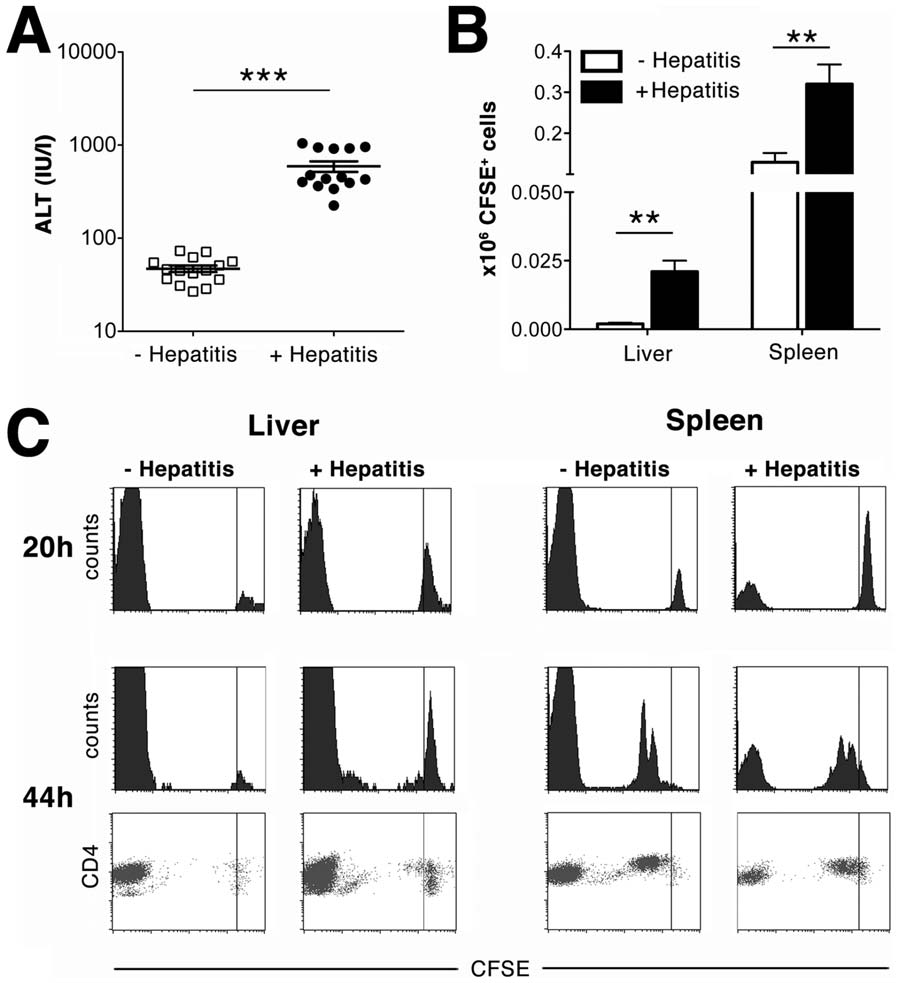
Priming of CD4 T-cells by endogenous antigen in the inflamed liver. (A) Eight million OT-I T-cells were transferred intravenously into TF-OVA mice (+ Hepatitis), or mice were left untreated (- Hepatitis). ALT levels were determined at day 6. Values from individual mice and mean ± SEM are depicted (*** p<0.0001 by Mann-Whitney test). (B) Four million CFSE-labeled OT-II T-cells were transferred intravenously into TF-OVA mice at day 6 after transfer of OT-I T-cells (+ Hepatitis) or into untreated TF-OVA mice (- Hepatitis). Non-parenchymal cells were isolated from liver and spleen analyzed for the presence of CFSE^+^ cells. The absolute number of CFSE^+^ cells in liver or spleen was determined 20 hours after transfer of OT-II T-cells. Cumulative results are depicted from n = 6 mice per group (mean ± SEM, ** p<0.005 by Student's t-test). (C) Mice were treated as in (B). Proliferation was analyzed 20 and 44 hours after transfer of OT-II T-cells. Events to the right of the vertical line represent the undivided population. Events at the far left of the plot represent unlabeled endogenous cells. Representative results are depicted from n = 6 mice in each group. All plots display data gated on CD4^+^Vα2^+^ cells.

Inflammation of the liver leads to up-regulation of MHC-II molecules on hepatocytes as well as to maturation of professional APCs. Both mechanisms could influence antigen presentation to CD4 T-cells and potentially allow their priming within the liver. To test whether this is the case, we induced hepatitis by transfer of OT-I T-cells prior to transfer of OT-II T-cells. At the peak of inflammation (i.e. 6 days after transfer of OT-I T-cells), we confirmed the presence of inflammation by measuring ALT-levels (Fig. 3A) before transferring carboxy-fluorescein succinimidyl-ester (CFSE)-labeled OT-II T-cells. Migration of OT-II T-cells to the liver and spleen was evaluated at 20 h, before proliferation of CD4 T-cells contributes to increased cell numbers. Significantly increased numbers of CFSE^+^ cells were observed in liver and spleen of mice suffering from hepatitis as compared to controls, demonstrating enhanced recruitment of CD4 T-cells to these organs (Fig. 3B). However, even under inflammatory conditions, we observed virtually no proliferation of transferred OT-II T-cells in the liver, as compared to the spleen (Fig. 3C). Thus, an inflammatory response does not promote antigen presentation sufficiently to allow priming of naïve CD4 T-cells by liver-derived antigen in the liver.

### CD4 T-cells primed by liver-derived antigen display deficient Th1-effector function

**Figure 4:**
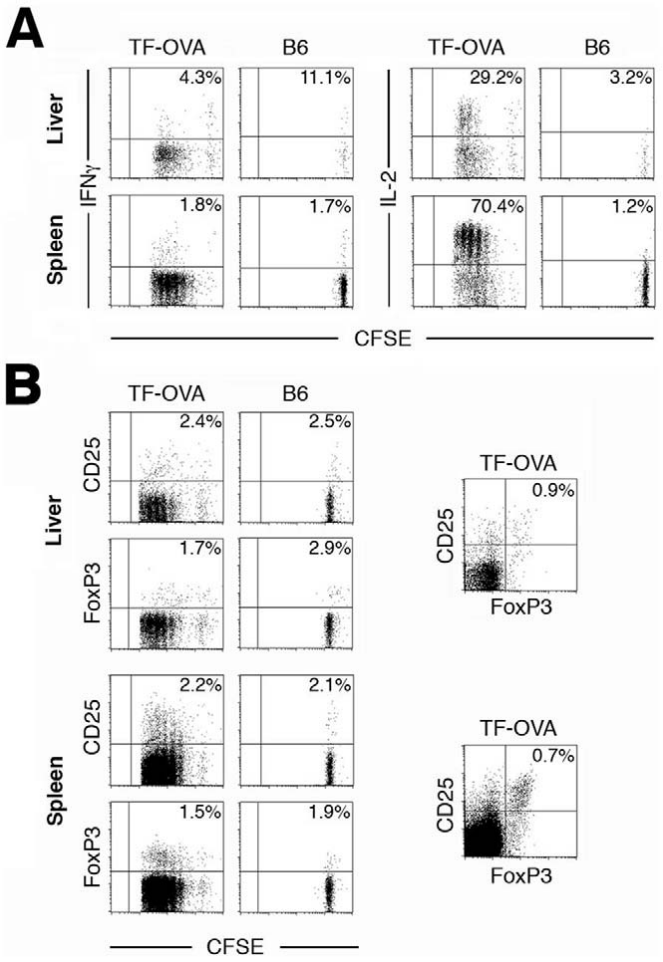
CD4 T-cells primed by liver-derived antigen display deficient Th1-effector function. (A) Four million CFSE-labeled OT-II T-cells were transferred intravenously into TF-OVA mice or into B6 control mice. After 68 hours, non-parenchymal cells were purified from liver and spleen and incubated *in vitro* with PMA/ionomycin. Production of Interferon-γ and IL-2 was analyzed after 4 hours. Representative results are depicted (n = 6). (B) Mice were treated as in (A), but cells were analyzed for CD25 and FoxP3 expression immediately after purification of cells from the indicated organs. Representative results are depicted from n = 6 mice in each group. All plots depict data gated on CD4^+^CFSE^+^ cells. CD25/FoxP3 plots on the right depict the frequency of CD25^+^FoxP3^+^ double positive cells.

Next we investigated whether naïve CD4 T-cells primed in TF-OVA mice exert effector function. To this end, CFSE-labeled OT-II T-cells were transferred into TF-OVA mice. To prove that activation is antigen-dependent, controls were carried out in B6 mice. Sixty-eight hours after transfer, lymphocytes were isolated from liver and spleen and re-stimulated *in vitro*. While re-stimulation induced considerable production of IL-2, very little Interferon-γ was produced by OT-II T-cells retrieved from liver or spleen (Fig. 4A).

To test whether OT-II cells primed in TF-OVA mice acquired a regulatory phenotype, we stained cells retrieved from liver and spleen for CD25 and FoxP3. Less than one percent of CFSE^+^CD4^+^ T-cells expressed CD25 and FoxP3 simultaneously (Fig. 4B), illustrating that induction of a regulatory phenotype in OT-II T-cells primed in TF-OVA mice is a rare event. Thus, activation of naïve CD4 T-cells by liver-derived antigen resulted in induction of cells capable of producing IL-2 but with a defective Th1-response, rather than in induction of complete anergy or a regulatory phenotype.

### Effector CD4 T-cells accumulate in the liver of TF-OVA mice

**Figure 5:**
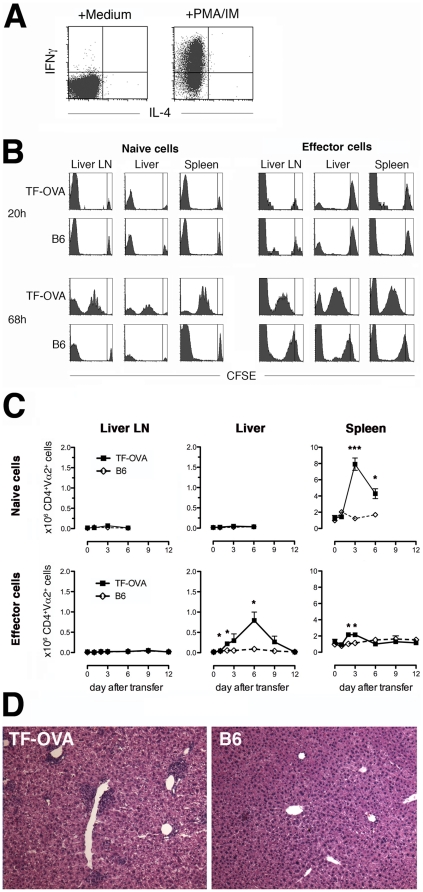
Effector CD4 T-cells accumulate in the liver of TF-OVA mice. (A) Effector OT-II T-cells with a Th1-phenotype were generated *in vitro* with the cognate peptide antigen in the presence of IL-12, Interferon-γ, and anti-IL-4 antibody. After 6 days in culture, cells were restimulated with PMA/ionomycin and stained for Interferon-γ and IL-4. (B) Four million CFSE-labeled naïve or effector OT-II T-cells were transferred into TF-OVA or B6 control mice. Non-parenchymal cells from the indicated organs were isolated after 20 or 68 hours, and analyzed for the presence of proliferating OT-II T-cells by CFSE dilution. All plots depict data gated on CD4^+^Vα2^+^ cells. Events to the right of the vertical line represent the undivided population. Events at the far left of the plot represent unlabeled endogenous cells. Representative results from n = 4–6 mice in each group are shown. (C) Cells from the indicated organs were isolated at the indicated days after transfer of 4 million naïve or effector OT-II T-cells, and numbers of CD4^+^Vα2^+^ cells were enumerated. Data shown are derived from n = 3–6 mice per group at each time point. Note the different scales for spleen and liver/liver lymph node (mean ± SEM; * p<0.05, *** p<0.001 by Student's t-test). (D) Liver sections from TF-OVA and B6 mice were stained with H&E 6 days after transfer of 4 million effector OT-II T-cells. Representative images from n = 6 mice per group (magnification 100x) are depicted.

Given the fact that naïve CD4 T-cells are not primed in the liver of TF-OVA mice, we tested whether effector CD4 T-cells migrate to the liver of TF-OVA mice and recognize their cognate antigen there. Effector OT-II cells with a Th1-phenotype were generated *in vitro.* Their ability to produce Interferon-γ was demonstrated after *in vitro* re-challenge with PMA/ionomycin (Fig. 5A). Twenty hours after transfer into TF-OVA or B6 control mice, effector OT-II T-cells were present in the liver in larger quantities than naïve T-cells, independently of antigen-expression (Fig. 5B, 20 h). Proliferation of these cells was observed 68 h after transfer in liver, spleen, and draining lymph nodes of TF-OVA mice (Fig. 5B, 68 h). A higher degree of CFSE-dilution was observed in TF-OVA mice than in B6 controls, indicating antigen-specific activation of the transferred effector OT-II T-cells. In contrast, no proliferation occurred after transfer of naïve OT-II T-cells into B6 mice.

Next we tested whether OT-II T-cells accumulate in the liver of TF-OVA mice after antigen recognition. To this end, 4 million naïve or effector OT-II T-cells were transferred into TF-OVA mice, and lymphoid organs and the liver were removed to enumerate OT-II T-cells at different time points. Since CFSE-dilution is complete after 3-4 days, the Vα2-chain of the OT-II T-cell receptor was used to estimate the number of OT-II T-cells. The increase of CD4^+^Vα2^+^ cells in livers of TF-OVA mice was much more pronounced than in livers of B6 control mice after transfer of effector T-cells (Fig. 5C). In the spleen, numbers of CD4^+^Vα2^+^ cells increased on days 2 and 3 but decreased to background levels at day six. In contrast, no accumulation of CD4 T-cells was observed in the liver after transfer of naïve CD4 T-cells, which accumulated solely in the spleen. In TF-OVA mice injected with effector OT-II T-cells, a dense lymphocytic infiltrate in the portal and periportal areas was observed 6 days after transfer (Fig. 5D).

Our results confirm that activated CD4 T-cells accumulate in the liver of TF-OVA mice and respond to antigen.

### Immune-mediated liver injury is amplified by effector but not naïve CD4 T-cells

**Figure 6:**
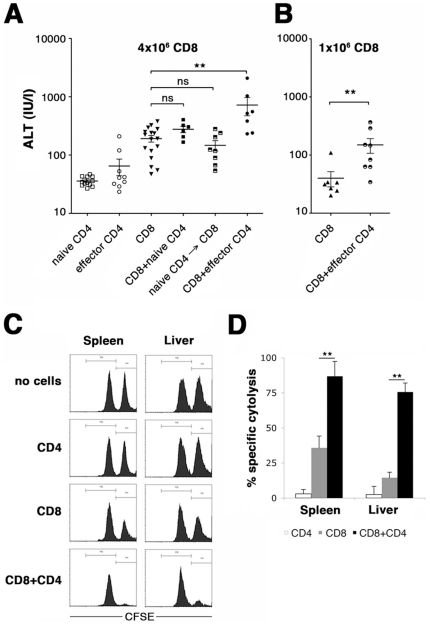
Hepatitis is amplified by effector but not naive CD4 T-cells. (A) Four million naïve (open squares) or effector (open circles) OT-II T-cells were transferred alone or together with 4 million naïve OT-I T-cells (filled squares and circles) into TF-OVA mice. Naïve OT-II T-cells were also transferred three days prior to transfer of OT-I T-cells to allow timely redistribution to the liver (half-filled squares). As a control, 4 million naïve OT-I T-cells (filled triangles) were transferred alone. Alanine aminotransferase levels were determined at day 5. Individual values and mean ± SEM are depicted (**p<0.005 by Mann-Whitney test). (B) One million naïve OT-I T-cells were transferred alone (filled triangles) or together with 4 million effector OT-II T-cells (half-filled circles) into TF-OVA mice. Alanine aminotransferase levels were determined at day 5. Individual values and mean ± SEM are depicted (**p<0.005 by Mann-Whitney test). (C) Four million effector OT-II T-cells were transferred alone (CD4) or together with 1 million naïve OT-I T-cells (CD8+CD4) into TF-OVA mice. As controls, 1 million naïve OT-I CD8 T-cells (CD8) were transferred alone or no cells were transferred (control). At day 6, equal numbers of CFSE^high^ SIINFEKL-pulsed and CFSE^low^ unpulsed B6 splenocytes were injected. After 5 hours cells from the indicated organs were analyzed for CFSE staining. Histogram blots depict data gated on CFSE-positive cells. (D) Antigen-specific cytolysis was calculated as described in methods. Cumulative results are depicted from n = 6 mice per group (mean ± SEM; ** p<0.005 by Mann-Whitney test).

We investigated the capacity of naïve and effector OT-II T-cells to induce liver damage. No significant increase in ALT-levels was observed after transfer of 4 million naïve or effector OT-II T-cells into TF-OVA mice (Fig. 6A), demonstrating that neither naïve nor effector CD4 T-cells are sufficient to induce hepatitis.

Since provision of help to CD8 T-cells is one of the main tasks of CD4 T-cells, we examined whether hepatitis induced by CD8 T-cells is augmented by addition of CD4 T-cells. The combination of naive OT-II and OT-I T-cells did not increase the severity of hepatitis compared to OT-I T-cells alone, even when transfer of CD4 T-cells preceded the transfer of CD8 T-cells by three days to ensure presence of CD4 T-cells in the liver at the time of CD8 T-cell priming. In contrast, the addition of effector OT-II T-cells to OT-I T-cells increased the severity of hepatitis, as judged from ALT-release. Three animals with very high ALT-levels died during the course of the experiment due to fulminant liver damage, while no animal died in any of the other groups. Extensive necrosis was present in the livers (data not shown). Therefore, we reduced the number of OT-I T-cells in the co-transfer experiment to such an extent that transfer of CD8 T-cells alone did not cause ALT-release (Fig. 6B). Co-transfer of effector OT-II T-cells with low numbers of OT-I T-cells lead to ALT-release, thus converting a sub-threshold damage elicited by CD8 T-cells alone to overt hepatitis when CD4 T-cell help is provided.

To prove that amplification of CD8 T-cell function by CD4 T-cell help is responsible for this observation, we determined the degree of cytolysis exhibited by OT-I T-cells in different settings. *In vivo* cytolytic activity was increased by the addition of effector OT-II T-cells (Fig. 6 C, D), suggesting that augmentation of CD8 T-cell activity is indeed responsible for the observed effect on the severity of liver damage.

## Discussion

The advent of mouse models of autoimmune hepatitis that do not rely on alloreactivity of T-cells [25], [26] provides experimental systems to study the role of T-cells in a model, in which professional as well as non-professional APCs contribute to T-cell priming [12], [13], [14]. These models have challenged the paradigm that CD8 T-cells primed by liver-derived antigen are rendered tolerant. Rather, effector function and hepatitis result from CD8 T-cell activation by liver-derived antigen when professional APCs are presenting the antigen. However, the effect of CD4 T-cells in such a setting remains elusive. We report here on the role of CD4 T-cells in an antigen specific model of immune-mediated liver injury.

We employed exogenous TCR-transgenic CD4 T-cells reactive to the neoantigen expressed in the liver and investigated their activation by different types of APCs *in vivo* and *in vitro*. We demonstrate that priming is achieved in the liver-draining lymph node and spleen by professional APCs but not by APCs within the liver. This finding is in sharp contrast to our findings on CD8 T-cells, which are efficiently primed within the liver. No proliferation was observed of CD4 T-cells primed *in vitro* on LSECs or *in vivo* in bone-marrow chimeras, in which only non-professional APCs are capable of presenting the antigen. Thus, LSECs are insufficient to prime CD4 T-cells by liver-derived antigen, at least under non-inflammatory conditions. Likewise, KCs and DCs isolated from the liver did not prime CD4 T-cells *in vitro*, and the absence of proliferating CD4 T-cells in the liver demonstrates that they do not activate CD4 T-cells by liver-derived antigen *in vivo,* either. Wuensch et al. also observed a lack of proliferation of CD4 T-cells in response to antigen introduced into hepatocytes by viral infection, albeit in a model in which the antigen is not cross-presented by professional APCs [27]. Therefore, direct comparison of the priming ability of specific APC populations on CD8 and CD4 T-cells is not possible. Our data indicate that the level of liver-derived antigen acquired by liver APCs *in vivo* suffices to stimulate CD8 T-cells. It is unclear whether the amount of antigen presented by the various APCs is too little to allow activation of CD4 T-cells or whether the low expression levels of MHC-II as well as of co-stimulatory molecules on liver APCs (reviewed in [28]), particularly KCs and LSECs, prevent activation. Observations obtained after addition of large amounts of exogenous antigen *in vitro* probably do not reflect the physiological situation *in vivo* and should therefore be interpreted with caution.

In a study by Lüth et al. [29], *in vivo* activation of CD4 T-cells by antigen expressed in the liver was studied. MBP-specific CD4 T-cells isolated from spleen and liver of mice transgenic for MBP expressed in hepatocytes proliferated, and a regulatory phenotype resulted, leading to suppression of experimental autoimmune encephalitis caused by autoreactive CD4 T-cells. The authors did not determine the type of APC and the site of activation leading to induction of the regulatory phenotype. In our experiments, only a small minority (<1%) of OT-II T-cells retrieved from liver or spleen displayed a regulatory phenotype as determined by expression of CD25 and FoxP3. However, we cannot exclude a regulatory function in the absence of FoxP3 expression, and even the small population of cells with a regulatory phenotype could suffice to suppress effector T-cells, as demonstrated by Lüth et al. Varying levels of antigen expression under the transgenic promoters or differences in the uptake or processing of the antigen by APCs may also contribute to the observed discrepancies.

Our findings have implications for the understanding of T-cell reactivity towards liver antigen. CD4 T-cells are not activated by liver-derived antigen in contrast to CD8 T-cells, possibly explaining why CD4 T-cell help is found infrequently in patients with viral hepatitis and exhaustion of the CD8 T-cell response results, leading to viral persistence. On the other hand, our data suggest that autoreactivity against liver antigen is most likely triggered by CD8 and not CD4 T-cells. Patients with autoimmune hepatitis display a mixed infiltrate of inflammatory cells in the liver, including CD8 and CD4 T-cells. In initial reports, CD4 T-cells were suggested as the main effector population causing liver damage in autoimmune hepatitis [30], [31], [32]. More recently it has become evident that CD8 T-cells play a dominant role, especially in the early phase of the disease [33], [34]. Our data support this notion.

In summary, our data add to existing evidence for a pivotal role of CD8 T-cells in the development of acute immune-mediated liver injury. Without CD8 T-cells, no liver damage is observed. Naïve CD4 T-cells are not primed within the liver by liver-derived antigen, neither by professional nor by non-professional APCs, but require activation in spleen or lymph nodes before re-locating to the liver. Effector CD4 T-cells are capable of infiltrating the liver but do not cause liver damage by themselves. While help provided by effector CD4 T-cells augments the severity of liver damage, CD4 T-cell help is dispensable for the induction of liver injury in the acute setting. CD4 T-cells likely play a more crucial role in maintaining the prolonged activation of CD8 T-cells observed in chronic hepatitis and in the formation of memory CD8 T-cells. Other cell-types such as NKT-cells [35] further regulate CD8 T-cell responses.

## Materials and Methods

### Animals and cells

All animals received humane care according to institutional criteria. All animal procedures were approved by the Landesamt für Gesundheit und Soziales, Berlin (registrations G0020/04 and G0191/09).

TF-OVA mice were described before [13]. Bone-marrow chimeras were generated by lethal irradiation of TF-OVA mice, followed by supplementation with C57BL/6 I-Aβ^−/−^ bone-marrow (Taconic, Hudson, NY). Mice were used for experiments after 6 weeks. Splenectomy was carried out under anesthesia with xylazin/ketamin, and mice were used for experiments after 2 weeks.

Naïve OT-I CD8 and OT-II CD4 T-cells were isolated from lymph nodes and spleen of OT-I [36] or OT-II [37] mice, respectively, using magnetic sorting (Miltenyi Biotec, Bergisch-Gladbach, Germany). Purity of preparations was above 90% for OT-I T-cells and above 80% for OT-II T-cells. For *in vitro* co-culture experiments and generation of CD4 effector cells, CD4^+^ T-cells were further selected for CD62L expression using magnetic beads (Miltenyi Biotec).

Liver APCs were isolated from TF-OVA mice by perfusion of the liver with 0.5 mg/ml collagenase in RPMI (Sigma, Taufkirchen, Germany). Fragmented livers were incubated for 20 min in digestion media at 37°C in an agitating incubator, passed through a nylon mesh, and briefly centrifuged at 300 rpm. Non-parenchymal cells were purified from the supernatant on a 42% Percoll gradient (Sigma, 2000 rpm, 20 min). Cells were separated into LSEC^+^ and LSEC^−^ fractions by magnetic separation with anti-CD146 beads (Miltenyi Biotec). The LSEC^+^ fraction was further sorted by FACS for CD31^+^CD45^−^ LSEC (both antibodies from ebioscience, San Diego, CA), resulting in >99% purity. From the LSEC^−^ fraction, KCs or DCs were isolated by FACS-sorting, resulting in >90% purity of F4/80^+^CD11b^+^ KCs or in >95% purity of CD11c^+^ DCs, respectively (antibodies from ebioscience and BD Biosciences, Heidelberg, Germany). Spleen DCs were obtained by FACS sorting of CD11c^+^ cells, resulting in >95% purity.

For *in vitro* co-culture experiments, 2×10^5^ APCs were cultured in 96 well flat bottom wells in 200 µl RPMI supplemented with 10% FCS, 1% penicillin/streptomycin, 1% gentamycin (Biochrom, Berlin, Germany), 1% L-Glutamin (Invitrogen, Karlsruhe, Germany), and 0.05 mM β-mercaptoethanol (Sigma), and allowed to settle for 24 h before addition of 1×10^5^ carboxy-fluorescein succinimidyl-ester (CFSE, Invitrogen, Karlsruhe, Germany) labeled T-cells [38]. Proliferation of OT-II or OT-I T-cells was analyzed after three days. OT-II and OT-I T-cells incubated alone were used as negative control. OT-II or OT-I T-cells incubated with irradiated spleen cells or specific APC populations and ovalbumin (100 mg/ml) served as positive control.

Effector CD4 T-cells were generated as described [24]. Briefly, irradiated B6 spleen cells were added at a 1∶3 ratio to purified OT-II T-cells, and 5 µg/mL OVA_323–339_ (ISQAVHAAHAEINEAGR, Institute of Biochemistry, Charité), 5 µg/ml anti-IL-4 (R&D Systems, Wiesbaden, Germany), 20 ng/mL Interferon-γ, and 5 ng/mL IL-12 (both Invitrogen) were added to RPMI supplemented with 10% FCS and β-mercaptoethanol. Cell cultures were split on day 3 and harvested on day 6. Dead cells were removed by a 24% NycoPrep density gradient (AxisShield, Oslo, Norway).

### Histology

For histology, livers were perfused with PBS and fixed for 24 h in 4% paraformaldehyd, followed by embedding in paraffin. 4 µm sections were stained with hematoxylin and eosin.

### FACS analysis

To isolate intrahepatic lymphocytes, livers were perfused with PBS/0.5% BSA, fragmented, passed through a 70 µm nylon mesh, and then treated as described above, before purification on a discontinuous 40/70% Percoll gradient. Antibodies against CD4, CD25, and Vα2 were from BD-Biosciences. For intracellular staining, cells were fixed and permeabilized using BD-Cytofix/CytopermTM (BD-Biosciences). Staining was performed with antibodies anti-Interferon-γ, anti-IL-2 (eBioscience), and anti-IL-4 (BD-Biosciences). Intracellular staining for FoxP3 was performed using the APC-FoxP3 staining set (eBioscience), according to the manufacturer's instructions. Cells were analyzed on a Becton Dickinson FacsCalibur using the CellQuest software.

### T-cell restimulation and *in vivo* cytolysis assay

For restimulation experiments, 4 million CFSE-labeled OT-II T-cells were transferred into TF-OVA mice. Mice were sacrificed at day 3, non-parenchymal cells were isolated from liver and spleen, cultured in complete RPMI, and activated in 20 nM phorbol 12-myristate 13-acetate/1 µM ionomycin (Sigma). After 1 hour, 2 µg/ml brefeldin-A (Sigma) was added, and cells were analyzed for intracellular cytokines after an additional 3 hours. Restimulation of *in vitro* generated effector cells was performed likewise.

For *in vivo* cytolysis assays, 4 million naïve or effector OT-II T-cells, 1 or 4 million naïve OT-I T-cells, or a combination of 1 or 4 million OT-I T-cells and 4 million naïve or effector OT-II T-cells were transferred into TF-OVA mice. Six days later, splenocytes from C57/BL6 mice were labeled in 7,5 µM or 0,75 µM CFSE and pulsed with 1 µg/ml SIINFEKL or left untreated for 1 hour, respectively. SIINFEKL-pulsed splenocytes and control splenocytes were mixed in equal numbers, and 8 million splenocytes were injected intravenously. As a control, splenocytes were injected into mice that had not received T-cells. After 5 hours, cells were isolated from lymphatic organs and liver and analyzed for CFSE staining. Specific lysis was calculated as follows: 100x[1-(%CFSE^lo^(control)/%CFSE^high^(control))/(%CFSE^lo^(OT-I)/%CFSE^high^(OT-I))].

### Alanine aminotransferase measurement

Blood was collected into separation tubes and sera were stored at −20°C before automatic analysis on a Roche modular analyzer (Grenzach-Wyhlen, Germany).

### Statistical analysis

Statistical analyses were performed with GraphPad Prism 5 software (GraphPad Software, San Diego, CA). The Student' s t test and the Mann-Whitney test were used as applicable.
